# Neuroprotection by Skimmianine in Lipopolysaccharide-Activated BV-2 Microglia

**DOI:** 10.3390/molecules28031317

**Published:** 2023-01-30

**Authors:** Folashade A. Ogunrinade, Victoria U. Iwuanyanwu, Satyajit D. Sarker, Olumayokun A. Olajide

**Affiliations:** 1 Department of Pharmacy, School of Applied Sciences, University of Huddersfield, Queensgate, Huddersfield HD1 3DH, UK; 2 Centre for Natural Products Discovery, School of Pharmacy and Biomolecular Sciences, Liverpool John Moores University, Byrom Street, Liverpool L3 3AF, UK

**Keywords:** natural products, skimmianine, neuroinflammation, neuroprotection, microglia, neurons

## Abstract

Skimmianine is a furoquinoline alkaloid which is found in the *Zanthoxylum* genus and also in other plants of the Rutaceae family. This study evaluated the effects of skimmianine on the production of pro-inflammatory mediators in LPS-activated BV-2 microglia. Cultured BV-2 cells were treated with skimmianine (10, 20 and 30 μM), followed by stimulation with LPS (100 ng/mL). Levels of TNFα and IL-6 in cell supernatants were measured using ELISA, while NO and PGE_2_ levels were evaluated with Griess assay and EIA, respectively. Western blotting was used to determine the protein expression of iNOS, COX-2, phospho-p65 and phospho-IκBα. Results showed that Skimmianine reduced LPS-induced elevated the secretion of TNFα, IL-6, NO, and PGE_2_, as well as the increased protein expression of iNOS and COX-2. Experiments to elucidate the mechanisms of the anti-neuroinflammatory activity of skimmianine revealed the significant inhibition of LPS-induced increased NF-κB-mediated luciferase activity. Pre-treatment with skimmianine also reduced LPS-induced the increased phosphorylation of NF-κB/p65 and IκBα proteins. Furthermore, skimmianine interfered with the binding capacity of NF-κB to consensus sites. Skimmianine pre-treatment protected HT-22 cells from toxicity induced by microglia-conditioned media, as well as increasing MAP-2 expression. The results of this study suggest that skimmianine inhibits neuroinflammation in LPS-activated microglia by targeting the NF-κB activation pathway. Skimmianine also produced neuroprotection against neurotoxicity induced by microglia-conditioned media.

## 1. Introduction

Mounting evidence continues to implicate neuroinflammation in the pathogenesis of neurodegenerative diseases [1], as well as neurological disorders such as depression [2] and epilepsy [3]. Neuroinflammation has also contributed to neurocognitive deficits of parasitic infections such as cerebral malaria [4,5]. The neuropathologies of viral infections such as HIV-associated neurocognitive disorder (HAND) and COVID-19 have been linked to brain inflammation [6,7,8,9]. Consequently, anti-inflammatory strategies are critical therapeutic modalities in these conditions.

Several studies suggest that some natural products inhibit neuroinflammation through mechanisms involving the inhibition of microglia activation and reduction of the release of pro-inflammatory cytokines from activated microglia by targeting one or more inflammatory signalling pathways in the microglia [10].

**Figure 1:**
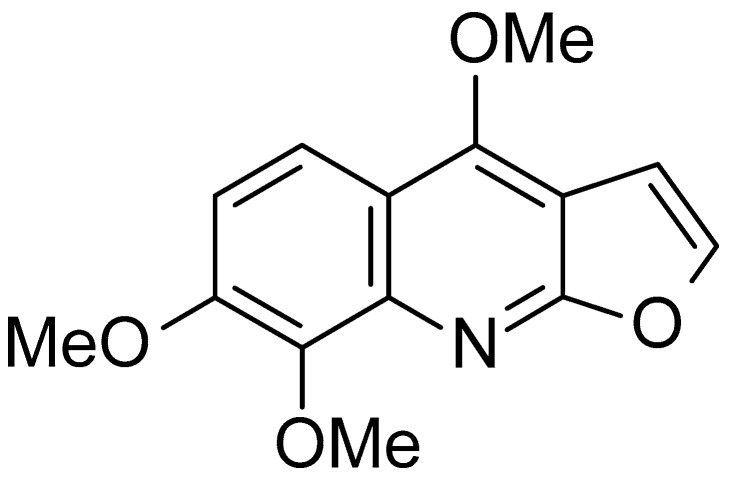
Structure of skimmianine.

Skimmianine {4, 7, 8-Trimethoxyfuro [2, 3-b] quinoline} (Figure 1) is the most abundant furoquinoline alkaloid found in the *Zanthoxylum* genus, and also in other genera of the family Rutaceae [11,12,13,14].

Some pharmacological activities have been attributed to skimmianine. Studies have shown that skimmianine isolated from *Zanthoxylum nitidum* and *Esenbeckia leiocarpa* produced anti-acetylcholinesterase activity in vitro [12,15].

Skimmianine isolated from *Ruta graveolens* produced anti-inflammatory activity against carrageenan-induced rat paw oedema, while mRNA expressions of TNFα and IL-6, as well as levels of PGE_2_ and TBARS were reduced [16]. Anti-inflammatory effects have also been reported for skimmianine isolated from *Tetradium ruticarpum* [17] and *Zanthoxylum avicennae* [18].

Reports have suggested that skimmianine isolated from the root barks of *Dictamnus dasycarpus* inhibited nitric oxide production in LPS-stimulated BV-2 cells [19]. However, this study did not investigate the effects of skimmianine on the production of other pro-inflammatory mediators in microglia.

In this study, we have evaluated the effects of skimmianine on the production of a wide array of pro-inflammatory mediators in LPS-activated microglia. We report the potential involvement of inhibiting activation of the NF-κB transcription factor in the anti-inflammatory activity of skimmianine. The impact of skimmianine on neuroinflammation-mediated toxicity to HT-22 mouse hippocampal neurons is also reported here.

## 2. Results

### 2.1. Skimmianine Did Not Affect the Viability of BV-2 Microglia

**Figure 2:**
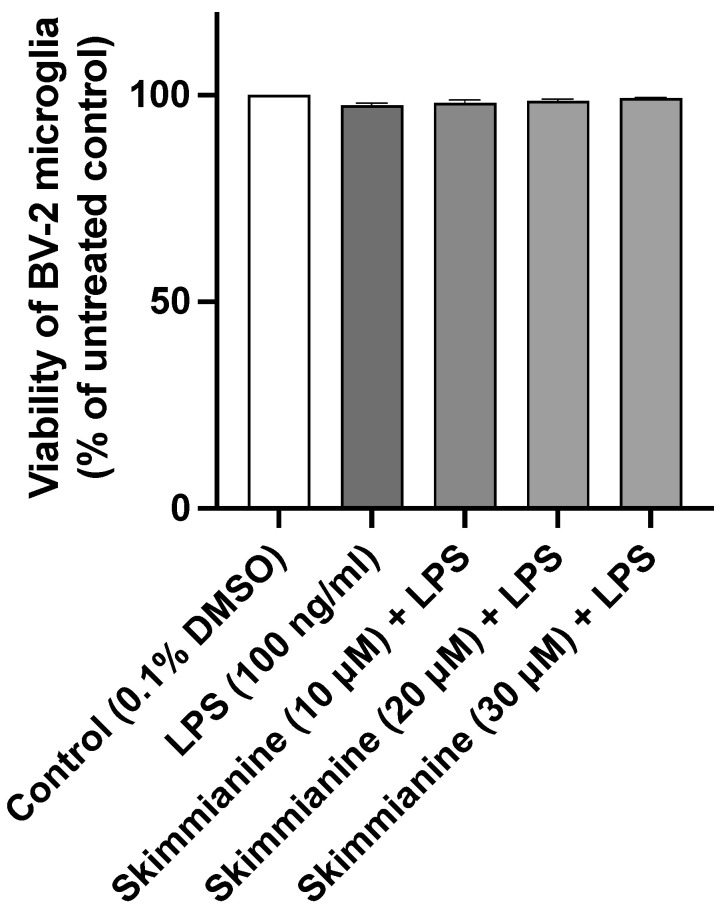
Skimmianine did not decrease the viability of BV-2 microglia cells. Pre-treatment with skimmianine (10, 20 and 30 µM) did not reduce the viability of BV-2 cells when stimulated with LPS (100 ng/mL) for 24 h. An MTT assay was used to determine the viability of BV-2 cells after treatment. All values are expressed as mean ± SEM for three independent experiments. A statistical analysis was performed using one-way ANOVA with a post hoc Dunnett’s test.

An MTT assay was used to measure the viability of BV-2 microglia cells following pre-treatment with skimmianine (10, 20 and 30 µM) for 30 min and then stimulation with LPS (100 ng/mL) for 24 h. The results in Figure 2 show that the viabilities of LPS-stimulated BV-2 microglia cells were not reduced in the presence of all concentrations of skimmianine used in this study.

### 2.2. Skimmianine Reduced Elevated Levels of TNFα and IL-6 in LPS-Activated BV-2 Microglia

**Figure 3:**
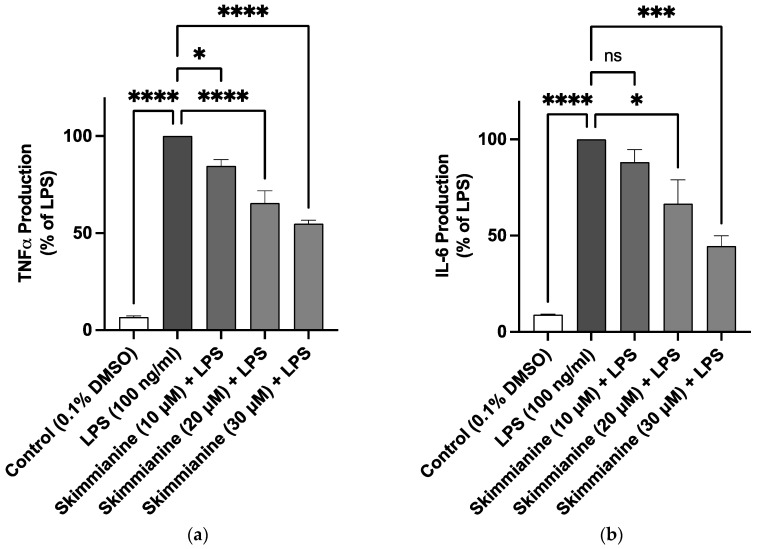
Skimmianine decreased TNFα and IL-6 production in BV-2 microglia cells stimulated with LPS. BV-2 cells were treated with or without skimmianine (10, 20 and 30 µM) and then stimulated with LPS (100 ng/mL) for 24 h. TNFα (**a**) and IL-6 (**b**) levels in cell culture supernatants were analysed using mouse ELISA kits. Values are mean ± SEM of at least three independent experiments. A statistical analysis was carried out using a one-way ANOVA with a post hoc Dunnett’s test. ns (not significant at *p* < 0.05); * *p* < 0.05; *** *p* < 0.001, **** *p* < 0.0001 in comparison with LPS stimulation.

The stimulation of BV-2 cells with LPS (100 ng/mL) induced a significant increase (*p* < 0.0001) in the production of TNFα when compared to untreated cells. However, pre-treatment with skimmianine at 10 µM produced ~15% reduction in TNFα production compared with LPS control. There was a further reduction (*p* < 0.001) in the levels of TNFα in the presence of 20 µM (~35% reduction) and 30 µM (~45% reduction) of the compound (Figure 3a).

Similarly, there was a significant elevation (*p* < 0.0001) in the levels of IL-6 in LPS-stimulated BV-2 cells compared to untreated cells. This increase was not significantly reduced by 10 µM of skimmianine. However, pre-treatment with skimmianine (20 µM and 30 µM) resulted in a significant reduction (*p* < 0.05) in the release of IL-6 in comparison to LPS-stimulated cells.

In experiments to determine the effects of skimmianine on IL-10 production, it was observed that the stimulation of BV-2 microglia with LPS (100 ng/mL) resulted in a significant (*p* < 0.001) reduction in the levels of IL-10. However, on pre-treating cells with skimmianine (10, 20 and 30 μM), a concentration-dependent increase in IL-10 production was observed (Supplementary Figure S1).

### 2.3. Effects of Skimmianine on iNOS-Mediated NO Production

**Figure 4:**
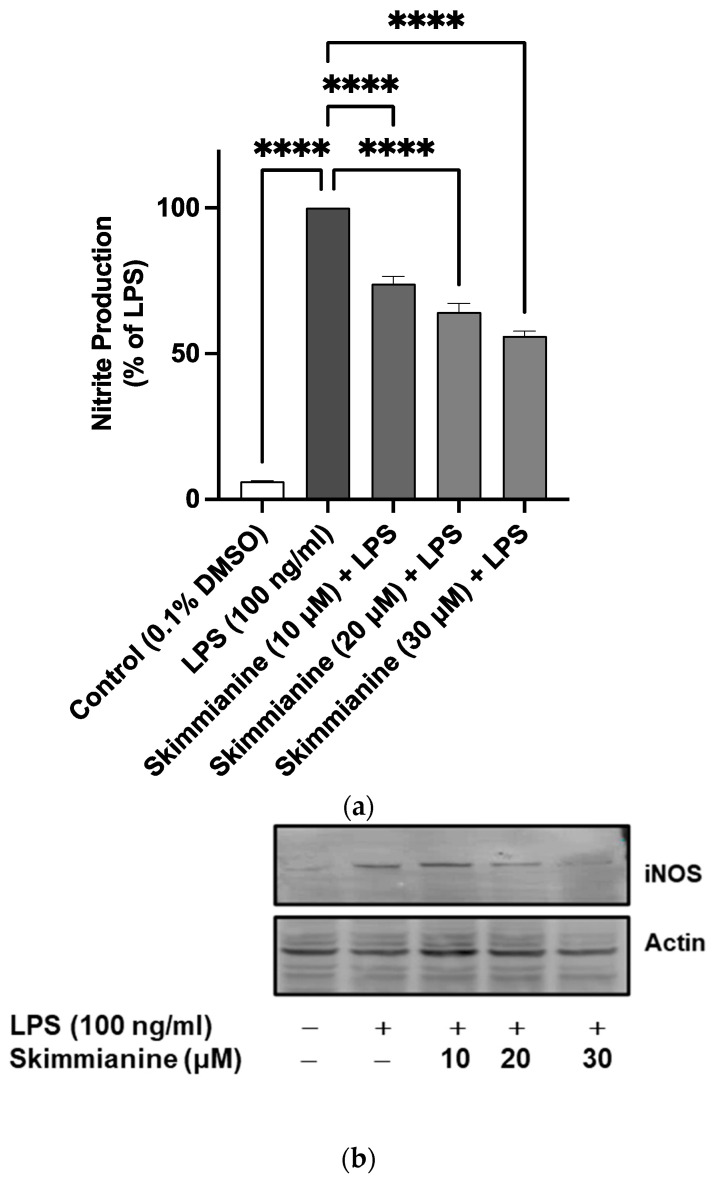

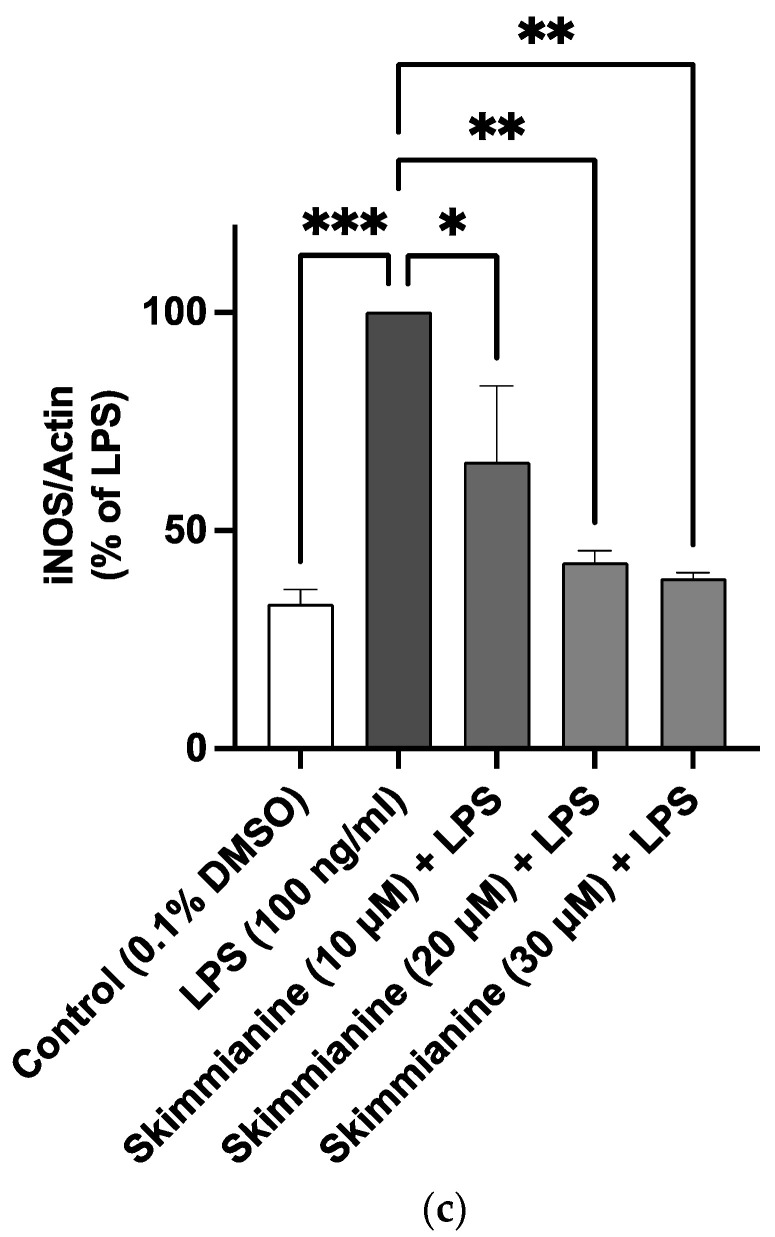
Skimmianine suppressed nitric oxide (NO) release (**a**) and inducible nitric oxide (iNOS) protein expression (**b**,**c**) in LPS-stimulated BV-2 cells. Cells were pre-treated with skimmianine (10, 20 and 30 µM) 30 min prior to stimulation with LPS (100 ng/mL) for 24 h. Cell culture supernatants were analysed for nitrite using the Griess assay, while western blotting was used to analyse iNOS protein expression in cell lysates. Values are mean ± SEM of at least three independent experiments. A statistical analysis was carried out using a one-way ANOVA with a post hoc Dunnett’s test. * *p* < 0.05; ** *p* < 0.01; *** *p* < 0.001; **** *p* < 0.0001 in comparison with LPS stimulation.

A significant (*p* < 0.0001) 16.6-fold increase in the levels of nitrite was observed following the stimulation of BV-2 cells with LPS (100 ng/mL) in comparison with unstimulated cells. Pre-treatment with skimmianine (10, 20 and 30 µM) prior to LPS stimulation resulted in a significant (*p* < 0.0001) reduction in nitrite production (Figure 4a).

LPS stimulation of BV-2 microglia caused a marked increase (*p* < 0.001) in iNOS protein expression compared to unstimulated cells. However, pre-treatment with 10, 20 and 30 µM resulted in ~44, ~59 and ~60% reductions in iNOS protein expression in comparison with LPS stimulation alone (Figure 4b,c).

### 2.4. Skimmianine Reduced PGE_2_ Production and COX-2 Protein Expression in LPS-Activated BV-2 Microglia

The results in Figure 4a show that LPS induced a significant increased (*p* < 0.0001) elevation in the levels of PGE_2_ when compared to unstimulated cells. However, treatment with skimmianine (10 µM) prior to LPS resulted in a significant (*p* < 0.001) reduction in PGE_2_ production. Further reductions in LPS-induced increased levels of PGE_2_ were observed in the presence of 20 µM (~34% reduction) and 30 µM (~49% reduction) of skimmianine.

**Figure 5:**
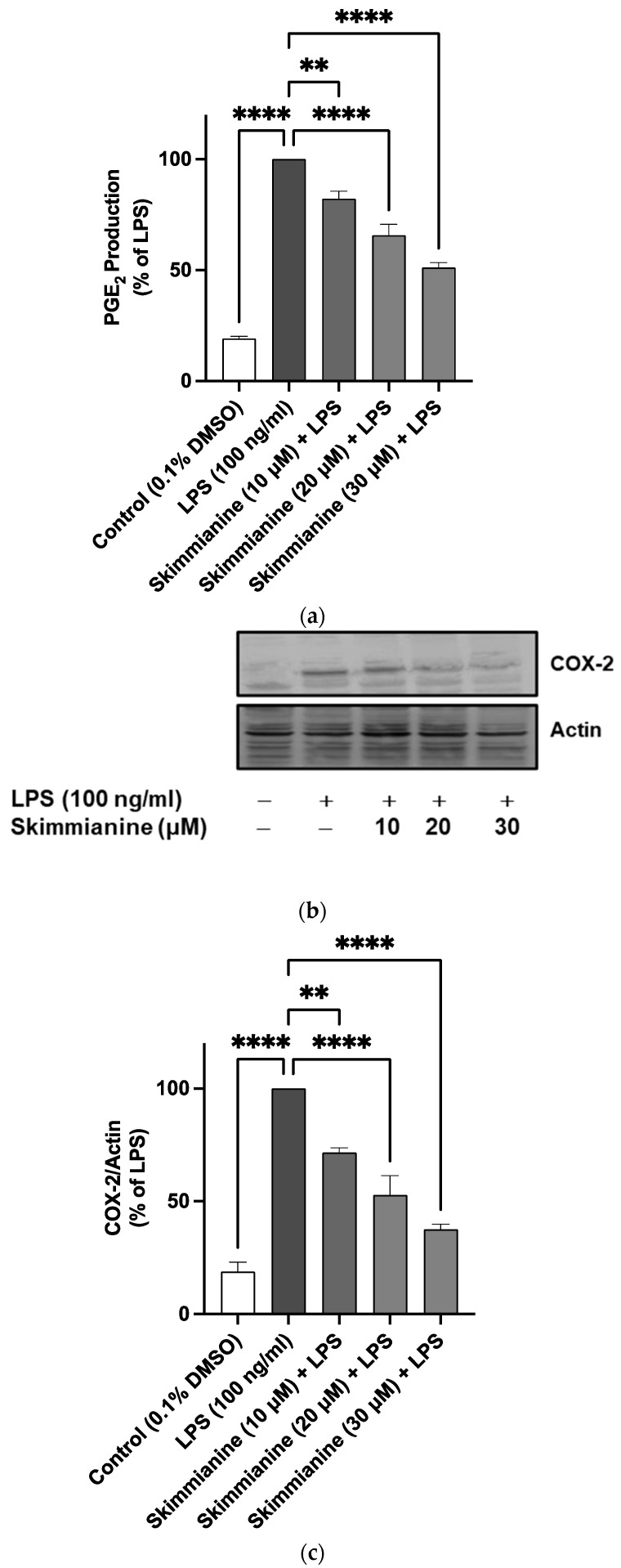
Skimmianine reduced PGE_2_ production (**a**) and COX-2 protein expression (**b**,**c**) in LPS-activated BV-2 microglia. Cultured microglia BV-2 cells were incubated with or without skimmianine (10, 20 and 30 µM) for 30 min before stimulation with LPS (100 ng/mL) for 24 h. Cell culture supernatants were analysed for PGE_2_ using EIA, while western blotting was used to analyse COX-2 protein expression in cell lysates. Values are mean ± SEM of at least three independent experiments. A statistical analysis was carried out using a one-way ANOVA with a post hoc Dunnett’s test. ** *p* < 0.01; **** *p* < 0.0001 in comparison with LPS stimulation.

LPS also induced a ~5.3-fold increase (*p* < 0.0001) in the expression of COX-2 protein. When cells were pre-treated with skimmianine (10, 20 and 30 µM), significant (*p* < 0.001) reductions in COX-2 protein expression were observed (Figure 5b,c).

### 2.5. Skimmianine Reduced LPS-Induced NF-κB-Mediated Gene Transcription in BV-2 Microglia

**Figure 6:**
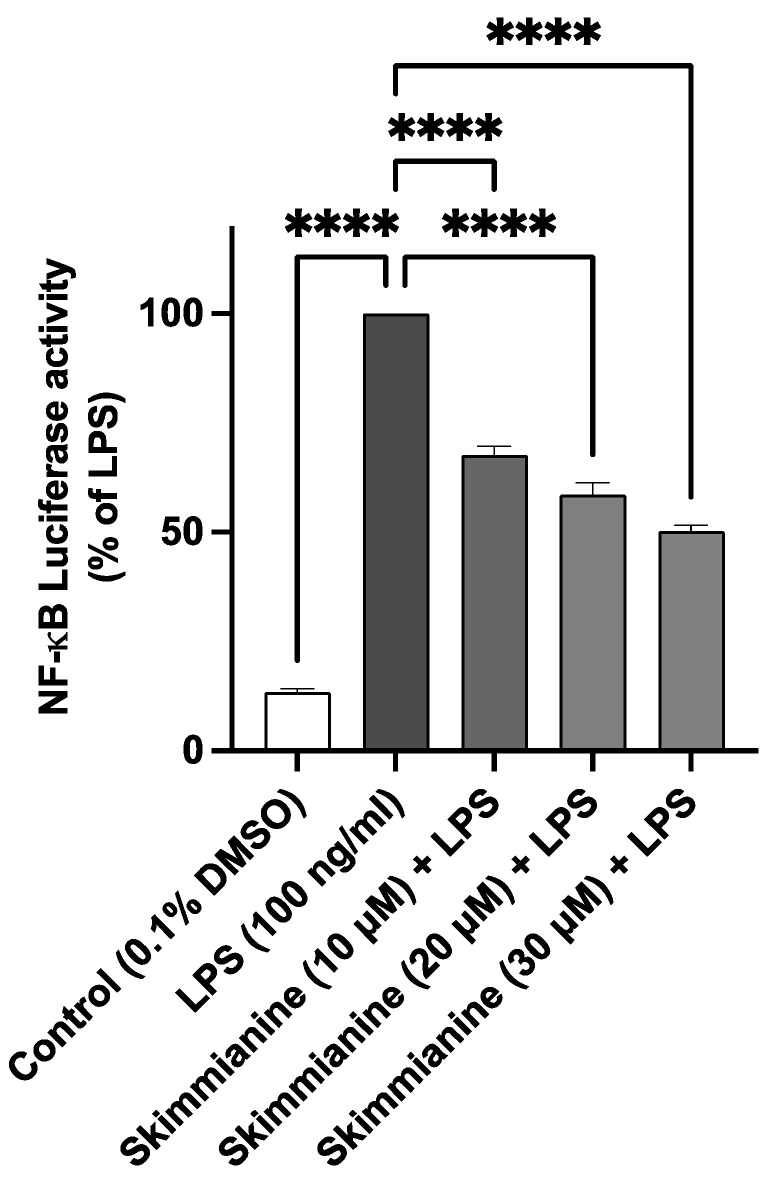
Skimmianine inhibits LPS-induced NF-κB-mediated gene expression. BV-2 microglia transiently transfected with a firefly luciferase gene driven by an NF-κB-regulated promoter were pre-treated for 30 min with skimmianine before stimulation with LPS (100 ng/mL) for 6 h. The cells were then lysed, and extracts were analysed for luciferase activity. A statistical analysis was carried out using a one-way ANOVA with a post hoc Dunnett’s test. Values are mean ± SEM of at least three independent experiments. **** *p* < 0.0001 in comparison with LPS stimulation.

Based on the outcome of experiments showing the inhibition of neuroinflammation by skimmianine through the reduction in the production of inflammatory mediators, its effect on NF-κB-mediated gene expression was evaluated. To achieve this, a plasmid construct bearing a luciferase reporter gene under the regulation of NF-κB was transiently transfected into BV-2 cells. The stimulation of transfected cells with LPS resulted in significant (*p* < 0.0001) activation of the NF-κB-driven luciferase activity, which was significantly (*p* < 0.0001) inhibited by skimmianine (10, 20 and 30 μM), as shown in Figure 6.

### 2.6. Skimmianine Inhibited Phosphorylation of IκB

**Figure 7:**
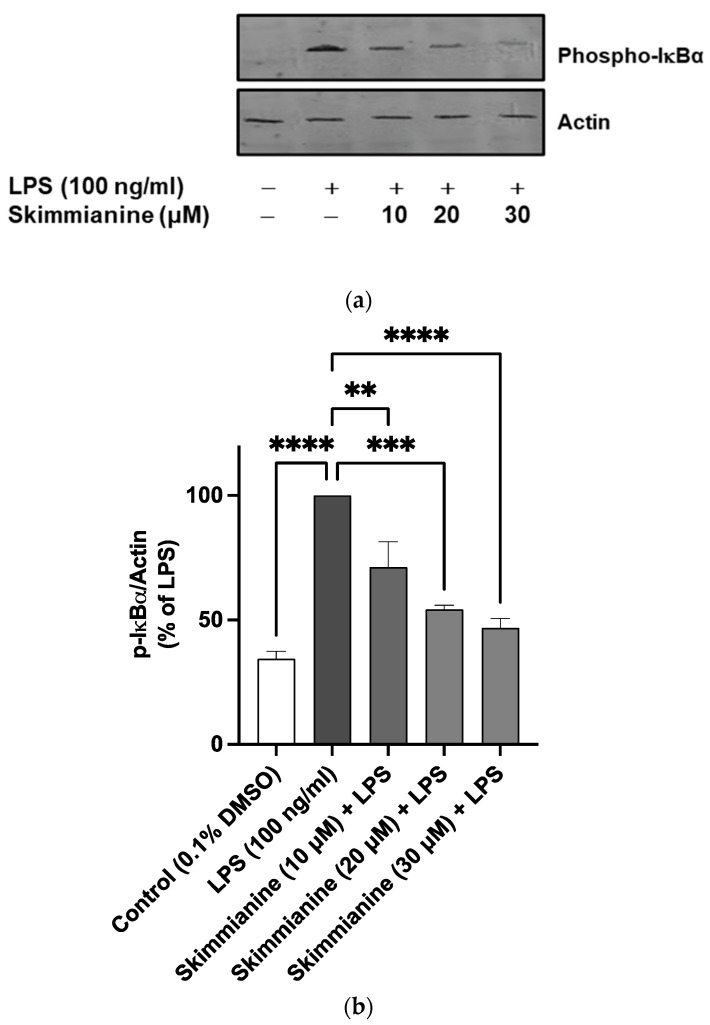
Skimmianine interferes with IκB phosphorylation. BV-2 microglia were pre-treated with skimmianine (10, 20 and 30 μM) for 30 min before they were stimulated with LPS (100 ng/mL). Cell lysates were subjected to a western blot analysis for phospho-IκBα (**a**). Densitometric analyses of the membranes were performed with Image J software (**b**). Values are mean ± SEM of at least three independent experiments. A statistical analysis was carried out using a one-way ANOVA with a post hoc Dunnett’s test. ** *p* < 0.01; *** *p* < 0.001; **** *p* < 0.0001 in comparison with LPS stimulation.

Encouraged by the marked inhibition of NF-κB-mediated gene transcription by skimmianine, the following experiments focused on whether skimmianine could affect the critical steps in the activation cascade of NF-κB. The results in Figure 7 show a significant (*p* < 0.0001) increase in the expression of phospho-IκBα following the stimulation of BV-2 cells with LPS (100 ng/mL). When cells were pre-treated with skimmianine (10, 20 and 30 μM), a significant and concentration-dependent reduction in protein expression of phospho-IκBα was observed.

### 2.7. Skimmianine Interferes with the Nuclear Translocation of p65/NF-κB

Observations of the inhibitory effects of skimmianine on phosphorylation and degradation of IκBα led to further studies to determine the impact of skimmianine on the translocation of p65/NF-κB subunit into the nucleus.

**Figure 8:**
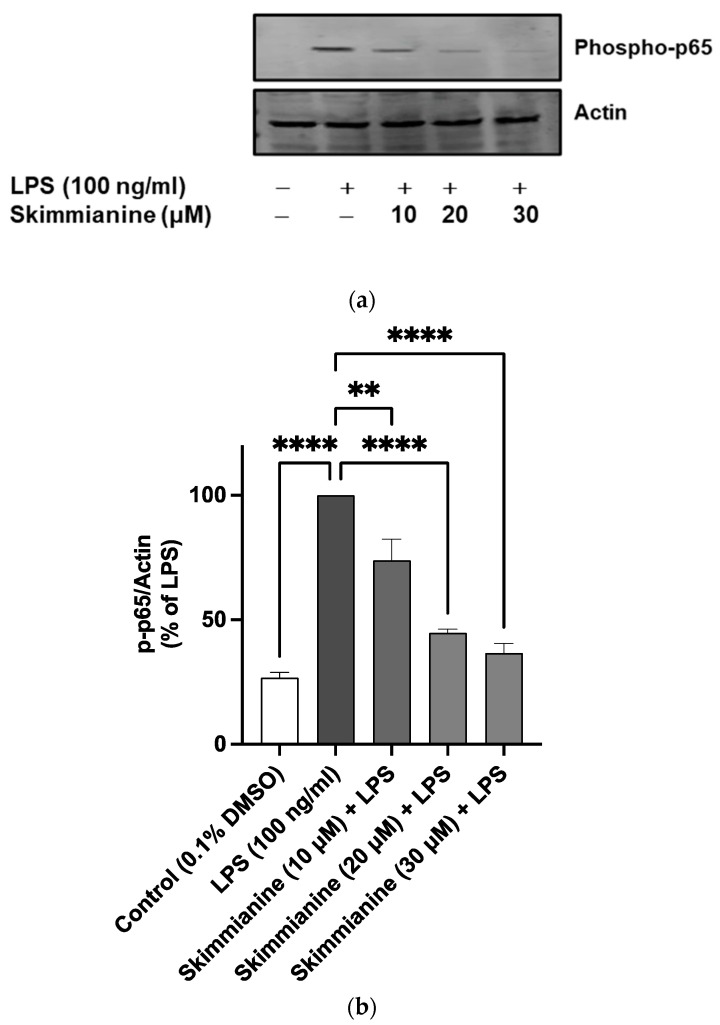
Skimmianine inhibits the nuclear translocation of p65/NF-κ in BV-2 microglia stimulated with LPS. BV-2 cells were stimulated with LPS (100 ng/mL) in the presence or absence of skimmianine (10, 20 and 30 µM) for 60 min. At the end of the incubation, cell lysates were analysed with western blotting (**a**). Densitometric analyses of the membranes were performed with Image J software (**b**). Values are mean ± SEM of at least three independent experiments. A statistical analysis was carried out using a one-way ANOVA with a post hoc Dunnett’s test. ** *p* < 0.05; **** *p* < 0.0001 in comparison with LPS stimulation.

Results from western blotting experiments revealed a significant (*p* < 0.0001) increase in phospho-p65 protein expression following the stimulation of BV-2 cells with LPS (100 ng/mL) for 60 min when compared with unstimulated cells. In the presence of 10, 20 and 30 μM skimmianine, the levels of phospho-p65 protein were reduced by 26%, 55% and 63%, respectively (Figure 8).

### 2.8. Skimmianine Interferes with the DNA Binding Capacity of NF-κB

Transcription factor assays were used to investigate the final step of NF-κB activation, which involves binding to its DNA consensus sequence.

**Figure 9:**
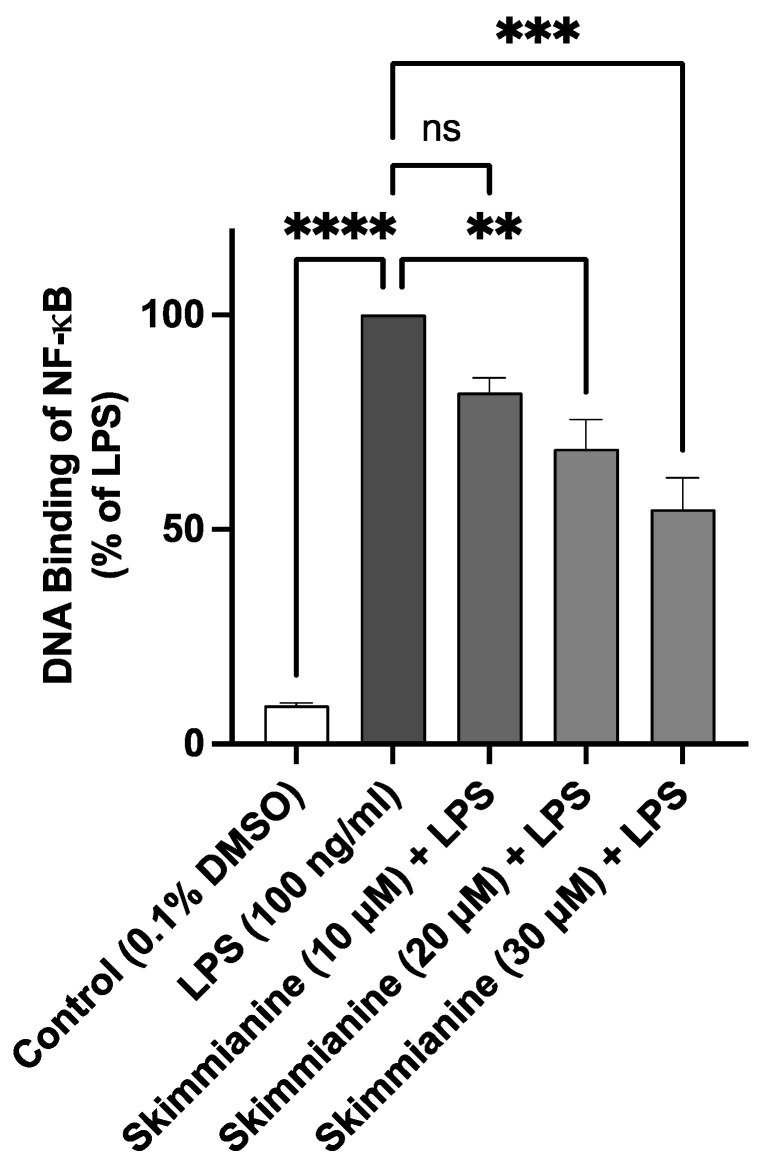
Skimmianine (20 and 30 µM) decreased the binding of NF-κB to consensus sites in LPS-stimulated BV-2 microglia. Nuclear extracts were added to wells pre-coated with the NF-κB consensus site (5′-GGGACTTTCC-3′), followed by the addition of the NF-κB p65 antibody. Values are mean ± SEM of at least three independent experiments. A statistical analysis was carried out using one-way ANOVA with a post hoc Dunnett’s test. ns (not significant at *p* < 0.05); ** *p* < 0.05; *** *p* < 0.001; **** *p* < 0.0001 in comparison with LPS stimulation.

Results in Figure 9 show an ~11-fold increase in the binding of NF-κB to consensus binding sites on stimulation of BV-2 microglia with LPS (100 ng/mL) compared with unstimulated cells. In the presence of skimmianine (10 µM), there was an insignificant (*p* < 0.05) reduction in the binding of NF-κB. However, on increasing the concentrations of skimmianine to 20 and 30 µM, the binding was significantly reduced (*p* < 0.001) compared with LPS stimulation alone.

### 2.9. Skimmianine Prevented LPS Neuroinflammation-Mediated Neurotoxicity in HT-22 Neurons

**Figure 10:**
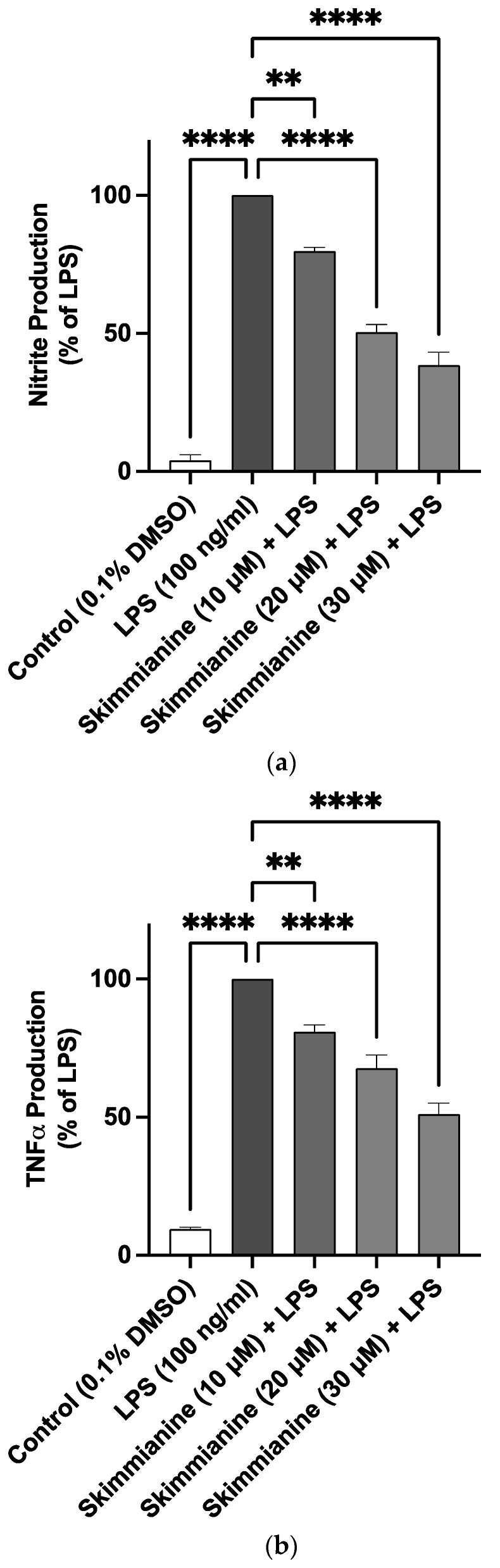

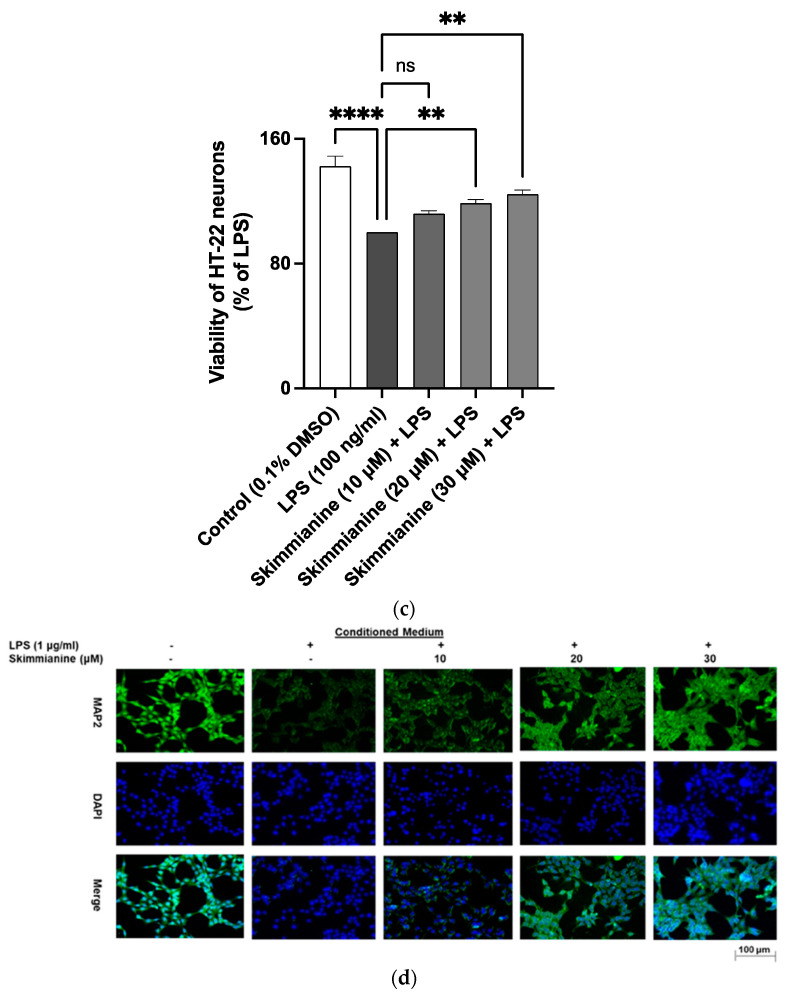
Skimmianine reduced neuroinflammation-mediated neurotoxicity in HT22 hippocampal neuronal cells. Cultured BV-2 cells were pre-treated with skimmianine for 30 min and stimulated with LPS (1 μg/mL) for 24 h. An ELISA was used to analyse the levels of nitrite (**a**) and TNFα (**b**) in conditioned media. An MTT viability assay showing the effect of incubation of conditioned media on the viability of HT-22 neurons (**c**). Immunofluorescence imaging showing the effect of conditioned media on MAP-2 expression in HT-22 cells (**d**). Values are mean ± SEM of at least three independent experiments. A statistical analysis was carried out using a one-way ANOVA with a post hoc Dunnett’s test. ns (not significant at *p* < 0.05); ** *p* < 0.05; **** *p* < 0.0001 in comparison with LPS stimulation.

Based on results showing the marked anti-inflammatory activity of skimmianine, experiments were conducted to determine whether the compound would affect LPS-conditioned medium-mediated neuronal toxicity. Analyses of conditioned medium from LPS-stimulated BV-2 microglia revealed a significant (*p* < 0.0001) elevation in levels of nitrite (~24-fold increase) and TNFα (~11-fold increase) in comparison to the conditioned medium from unstimulated cells (Figure 10a,b). The levels of nitrite in the conditioned medium from cells pre-treated with skimmianine (10, 20 and 30 µM) were significantly (*p* < 0.001) reduced in comparison to LPS-stimulated cells (Figure 10a). Similarly, pre-treatment with skimmianine resulted in a significant reduction in TNFα levels in the conditioned medium (Figure 10b).

The MTT cell viability assay revealed that exposure of HT-22 neurons to conditioned medium from LPS-stimulated BV-2 microglia resulted in a ~29% reduction in neuronal viability (Figure 10c). However, the incubation of conditioned medium from BV-2 cells pre-treated with skimmianine (20 and 30 µM) before LPS stimulation resulted in a significant (*p* < 0.01) improvement in neuronal cell viability (Figure 10c).

The immunofluorescence imaging of HT-22 neurons incubated with LPS-stimulated BV-2 microglia conditioned medium reveals a decrease in the expression of the MAP-2 protein. On the other hand, MAP-2 expression was increased in HT-22 cells incubated with conditioned medium from BV-2 microglia pre-treated with skimmianine (10, 20 and 30 µM) before stimulation with LPS (1 μg/mL) (Figure 10d).

## 3. Discussion

The search for natural product inhibitors of neuroinflammation continues to be an important strategy in ameliorating neuronal damage associated with neurodegenerative and neurological disorders. Several studies have linked the activation of microglia in neuroinflammation to neuronal cell death and symptoms of neurodegenerative disorders such as Alzheimer’s disease [20]. An important consequence of microglial activation is the release of cytokines such as IL-6 and TNFα, which leads to neuronal damage [21]. The results of this study revealed that skimmianine reduced elevated levels of the pro-inflammatory cytokines TNFα and IL-6 in LPS-activated BV-2 microglia, suggesting that the compound inhibits neuroinflammation. Skimmianine has been previously reported to produce anti-inflammatory activity against carrageenan-induced rat paw oedema by reducing TNFα and IL-6 [16]. This is, however, the first report showing a reduction of the levels of these cytokines in vitro, specifically in microglial cells. The anti-inflammatory activity of skimmianine was confirmed in experiments showing its effect in increasing the production of the anti-inflammatory cytokine IL-10.

It is well established that iNOS-mediated excessive production of nitric oxide (NO) in the activated microglia results in neurotoxicity [22,23,24,25]. The excessive production of NO and iNOS protein expression was shown in this study to be inhibited by skimmianine in BV-2 microglia activated with LPS. Yoon et al. [19] previously reported the inhibition of nitric oxide by skimmianine and other alkaloids isolated from root barks of *Dictamnus dasycarpus* in (LPS)-stimulated BV-2 cells. However, the study by Yoon et al. did not report the effects of skimmianine on iNOS protein expression. The similarity between our results and those reported by Yoon et al. appears to suggest that plants belonging to the Rutaceae family may contain a number of biologically active constituents which warrant investigation as potential neuroprotective natural products. Interestingly, plants in the Rutaceae family such as *Citrus aurantium*, *Citrus medica*, *Citrus medica*, *Citrus sinensis*, *Ruta chalepensis*, *Ruta graveolens*, *Zanthoxylum acanthopodium*, *Zanthoxylum bungeanum*, and *Zanthoxylum zanthoxyloides* have been reported to produce anti-inflammatory activity in vivo and in vitro [14,26,27,28,29,30,31,32].

Several reports have linked the production of prostaglandin E2 (PGE_2_) by activated microglia to neuronal death [33,34,35]. In fact, elevated levels of PGE_2_ and cyclooxygenase-2 (COX-2) have been reportedly linked with cognitive impairment in mice [36] as well as in Alzheimer’s disease patients [37,38]. Studies to further evaluate the anti-inflammatory effects of skimmianine in the microglia showed that the compound reduced the elevated production of PGE_2_ and increased the expression of COX-2 protein in BV-2 microglia activated with LPS.

In this study, the inhibition of neuroinflammation by skimmianine was established following pre-treatment with the compound for 30 min, followed by stimulation with LPS for 24 h. However, a study reported by Kučić et al. [39], showed that the inhibition of neuroinflammation was in fact possible by stimulating BV-2 microglia prior to treatment with low concentrations of naltrexone. It would therefore be interesting if future studies could determine whether reversing the sequence of skimmianine treatment and LPS stimulation would affect the anti-inflammatory activity of the compound.

Encouraged by the results showing the inhibition of neuroinflammation by skimmianine of its ability to reduce the production of multiple pro-inflammatory mediators in LPS-activated BV-2 microglia, and since NF-κB is responsible for regulating the expression of genes encoding these mediators, the effects of the compound on NF-κB-mediated gene expression was evaluated. The results showed that skimmianine produced the effective inhibition of NF-κB-mediated luciferase transcription, suggesting that the compound produces anti-inflammatory activity through mechanisms involving NF-κB activation. This observation led to experiments which focused on determining whether skimmianine could affect the critical steps in the activation cascade of NF-κB. One of the early steps in the responses of microglia to LPS is the phosphorylation and degradation of IκBα, which complexes NF-κB in the cytoplasm under resting conditions. It was shown that skimmianine interferes with the phosphorylation of IκBα, as well as the phosphorylation of p65/NF-κB, which are steps that are upstream of NF-κB mediated gene expression.

The results of transcription factor (DNA binding) experiments showed that, in addition to its effect on cytoplasmic processes, skimmianine directly acted on the DNA binding step of activated NF-κB and thus interfered with the transcription of pro-inflammatory proteins such as TNFα, IL-6, iNOS and COX-2.

While this is the first report of NF-κB-mediated anti-inflammatory activity by skimmianine, several extracts and compounds isolated from plants in the Rutaceae family have been reported to produce anti-inflammatory activity through mechanisms involving the inhibition of NF-κB activation [14,27,40,41,42,43,44,45].

Once the anti-neuroinflammatory activity was established, it was hypothesised that skimmianine could indirectly inhibit neurotoxicity by regulating microglial neuroinflammation. Consequently, the toxicity of BV-2 microglia-conditioned media on HT-22 neurons was evaluated. Using this approach, skimmianine was shown to produce a neuroprotective effect, which was confirmed by an increase in the expression of the neuronal microtubule-associated protein 2 (MAP-2) protein.

In the current study, we showed that skimmianine inhibits neuroinflammation in LPS-activated microglia by targeting the NF-κB activation pathway. Skimmianine was also shown to prevent neurotoxicity caused by microglia-conditioned media.

## 4. Materials and Methods

### 4.1. Materials

Skimmianine was purchased from PhytoLab GmbH & Co. KG (Vestenbergsgreuth, Germany). Lipopolysaccharide (LPS) solution from *Salmonella enterica* serotype Typhimurium was purchased from Sigma-Aldrich (Gillingham, UK).

### 4.2. Cell Culture

The BV-2 mouse microglia cell line (ICLCATL03001) was purchased from the Interlab Cell Line Collection (Banca Biologica e Cell Factory, Genova, Italy) and cultured in Roswell Park Memorial Institute (RPMI) medium supplemented with 10% foetal bovine serum, L-glutamine (2 mM) and penicillin (100 U/mL) + streptomycin (100 μg/mL).

The HT-22 mouse hippocampal neurons were a gift from Dr Jeff Davis (Swansea University, UK). The cells were cultured in DMEM supplemented with 10% foetal bovine serum, L-glutamine (2 mM) and penicillin (100 U/mL) + streptomycin (100 μg/mL). All cells were maintained at 37 °C in 5% CO_2_.

### 4.3. Determination of BV-2 Cell Viability

Cultured BV-2 cells were treated with skimmianine (10, 20 and 30 μM) for 30 min and then stimulated with LPS (100 ng/mL) for 24 h. After the incubation period, the cell culture medium was removed and replaced with MTT solution (5 mg/mL), followed by incubation for 4 h. Thereafter, 150 μL of MTT solution was removed from each well and replaced with 150 μL DMSO. Formazan crystals were dissolved by shaking the plate on a rocker, and absorbance was read in a Tecan Infinite F50 microplate reader at a wavelength of 570 nm.

### 4.4. ELISA for TNFα, IL-6 and IL-10

Cultured BV-2 cells (2 × 10^5^ cells/mL) were treated with skimmianine (10, 20 and 30 μM) for 30 min, followed by stimulation with LPS (100 ng/mL) for 24 h. Levels of TNFα, IL-6 and IL-10 in culture supernatants were measured with mouse TNFα and IL-6 ELISA kits (Biolegend), respectively, according to the manufacturer’s instructions.

### 4.5. Griess Assay

Cultured BV-2 cells (2 × 10^5^ cells/mL) were treated with skimmianine (10, 20 and 30 μM) for 30 min, followed by stimulation with LPS (100 ng/mL) for 24 h. Thereafter, the supernatants were analysed for levels of nitrite using the Griess reagent kit (Promega, Chilworth, UK). Briefly, 50 μL of sulphanilamide was added to 50 μL of culture supernatants and incubated in the dark for 10 min at room temperature. Afterwards, 50 μL of N-1-naphthyl ethylenediamine solution was added and incubated in the dark for another 10 min at room temperature. The absorbance was read in a Tecan Infinite F50 microplate reader at a wavelength of 540 nm.

### 4.6. Enzyme Immunoassay for PGE_2_

Culture supernatants from skimmianine (10, 20 and 30 μM)-treated and LPS (100 ng/mL)-stimulated BV-2 microglia were analysed for levels of PGE_2_ using an enzyme immunoassay kit (Arbor Assays). Briefly, 100 µL of samples and standards were added to wells pre-coated with goat anti-mouse IgG. Subsequently, 25 µL of PGE_2_ conjugate and PGE_2_ antibody were added and incubated at room temperature for 2 h with shaking at 250 rpm. Thereafter, the wells were washed to remove unbound proteins. This was followed by the addition of 100 µL 3, 3′, 5, 5′-Tetramethylbenzidine (TMB) substrate to each well and incubation for 30 min at room temperature. The absorbance was measured in a microplate reader (Infinite F50, Tecan, Männedorf, Switzerland) at a wavelength of 450 nm following the addition of a stop solution.

### 4.7. Western Blotting

BV-2 cells were treated with skimmianine (10, 20 and 30 μM) for 30 min, followed by stimulation with LPS (100 ng/mL) for different time points. Immunoblotting was carried out on 20–40 μg of cell lysates which were subjected to sodium dodecyl sulfate-polyacrylamide gel electrophoresis (SDS-PAGE), followed by transfer onto polyvinylidene fluoride (PVDF) membranes (Millipore, Burlington, MA, USA). The membranes were incubated with blocking buffer at room temperature for 1 h and further incubated with primary antibodies overnight at 4 °C. The primary antibodies used were rabbit anti-COX-2 (1:1000; Abcam, Cambridge, UK), rabbit anti-iNOS (1:1000; Cell Signaling Technology, Danvers, MA, USA), rabbit anti-phospho-IκBα (1:500; Santa Cruz Biotechnology, Dallas, TX, USA), rabbit anti-phospho-p65 (1:500; Abcam), and rabbit anti-actin (1:1000; Sigma). Thereafter, membranes were washed in tris-buffered saline + Tween 20 (TBS-T), followed by incubation with Alexa Fluor 680 goat anti-rabbit secondary antibody (1:10,000; Thermo Scientific, Waltham, MA, USA) at room temperature for 1 h. Blots were detected using a Licor Odyssey Imager. All Western blot experiments were carried out at least three times, and blots were quantified using Image J software (Version 9).

### 4.8. Transient Transfection and NF-κB Reporter Gene Assay

BV-2 microglia were cultured to 60% confluence. Cells were then transfected with Cignal NF-κB luciferase reporter (Qiagen, Venlo, Netherlands) using magnetofection (OZ Biosciences, San Diego, CA, USA), as previously described [9], and incubated for 20 h at 37 °C. At the end of the incubation period, cells were stimulated with LPS (100 ng/mL) in the presence or absence of skimmianine (10, 20 and 30 µM) for 6 h. Luciferase activity was determined with a Dual-Glo luciferase assay kit (Promega, UK). Luminescence was measured using a POLARstar Optima microplate reader (BMG Labtech, Ortenberg, Germany).

### 4.9. NF-κB Transcription Factor Assay

The DNA binding capacity of NF-κB following activation by LPS was quantitatively evaluated using the ELISA-based TransAM^®^ NF-κB transcription factor kit (Active Motif, Waterloo, Belgium). This kit contained a 96-well plate to which an oligonucleotide containing the NF-κB consensus site (5′-GGGACTTTCC-3′) has been immobilised. After treating BV-2 microglia with skimmianine (10, 20, and 30 µM) for 30 min, the cells were stimulated with LPS (100 ng/mL) for 1 h. Nuclear extracts were analysed in DNA binding assays according to the manufacturer’s instructions.

### 4.10. BV-2 Microglia Conditioned Medium-Mediated Neurotoxicity

Excessive production of pro-inflammatory mediators such as TNFα and IL-6 in activated microglia cells induces damage to surrounding neurons. Cultured BV-2 microglia seeded in a 6-well plate at a concentration of 2 × 10^5^ cells/mL were treated with skimmianine at 10, 20 and 30 μM 30 min prior to stimulation with LPS (1 μg/mL) for a further 24 h. At the end of the incubation, supernatants were collected (conditioned medium). Levels of NO and TNFα were analysed in the conditioned media to confirm their secretion from LPS-activated BV-2 microglia.

HT-22 neuronal cells were seeded in a 96-well plate at a concentration of 2 × 10^5^ cells/mL and incubated until the cells reached confluence. At the end of the incubation, the culture medium was carefully removed from each well and replaced with 200 µL of conditioned medium from activated BV-2 cells, followed by incubation for 24 h. Thereafter, an MTT assay was used to determine the viability of HT-22 neuronal cells.

The effect of skimmianine on the expression of MAP-2 protein in conditioned medium-exposed HT-22 cells was observed by immunofluorescence. Briefly, cells were fixed with ice-cold methanol (100%) for 15 min at −20 °C. The cells were then incubated overnight at 4 °C with an anti-MAP-2 antibody (1:100). This was followed by incubation with Alexa Flour 488-conjugated donkey anti-rabbit IgG secondary antibody (Life Technologies, Carlsbad, CA, USA; 1:100). Cells were counterstained with 50 nm of DAPI (Invitrogen; 50 nM) for 5 min. Fluorescence images were acquired using an EVOS^®^ FLoid cell imaging system (Life Technologies).

### 4.11. Statistical Analysis

The values of all the results are represented as the mean ± SEM of at least 3 independent experiments. Data were analysed using a one-way analysis of variance followed by a post hoc Dunnett’s test. Statistical differences of *p*-value less than 0.05 (*p* < 0.05) were considered significant. Data were analysed using GraphPad Prism software (version 9).

## Data Availability

The data presented in this study are available on request from the corresponding author.

## Supplementary Materials

The following supporting information can be downloaded at: https://www.mdpi.com/article/10.3390/molecules28031317/s1, Supplementary Figure S1: Skimmianine increased IL-10 production in BV-2 microglia cells stimulated with LPS.

## Sample Availability

Samples of the compounds are available from the authors.
