# Supplementary material for: Neuroprotection by Skimmianine in Lipopolysaccharide-Activated BV-2 Microglia
Source: Molecules. 2023 Jan 30;28(3):1317. doi: 10.3390/molecules28031317 (PMC9920223; doi:10.3390/molecules28031317)
Supplement: Supplementary file 1 [file molecules-28-01317-s001.zip › molecules-2167009-supplementary.pdf]

Supplementary

Neuroprotection by skimmianine in lipopolysaccharide-activated BV-2 microglia

Folashade A Ogunrinade [1], Victoria U Iwuanyanwu [1], Satyajit D Sarker [2] and Olumayokun A Olajide [1,*]

1 Department of Pharmacy, School of Applied Sciences, University of Huddersfield, Queensgate, Huddersfield, HD1 3DH, United Kingdom
2 Centre for Natural Products Discovery, School of Pharmacy and Biomolecular Sciences, Liverpool John Moores University, Byrom Street, Liverpool L3 3AF, United Kingdom
* Correspondence: o.a.olajide@hud.ac.uk; Tel.: +44 (0) 1484 472735.

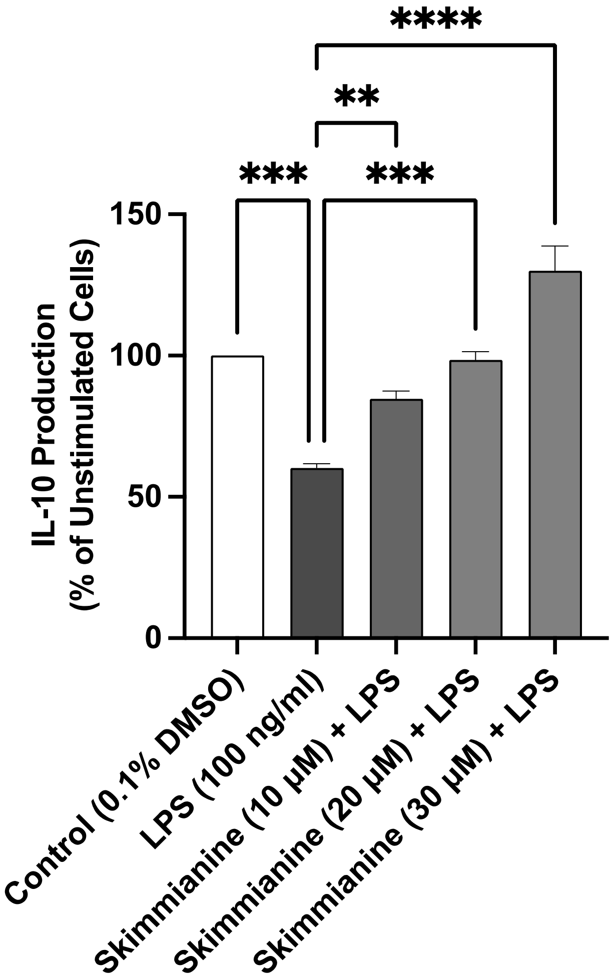

Supplementary Figure S1. Skimmianine increased IL-10 production in BV-2 microglia cells stimulated with LPS. BV-2 cells were treated with or without skimmianine (10, 20 and 30 μM) and then stimulated with LPS (100 ng/mL) for 24 h. IL-10 levels in cell culture supernatants were analysed using mouse ELISA kits. Values are mean

± SEM of at least 3 independent experiments. Statistical analysis was carried out using one-way ANOVA with post hoc Dunnett test. ** $p < 0.01$; *** $p < 0.001$, **** $p < 0.0001$ in comparison with LPS stimulation.
